# Allosteric Inhibition of the SARS‐CoV‐2 Main Protease: Insights from Mass Spectrometry Based Assays**

**DOI:** 10.1002/anie.202010316

**Published:** 2020-10-15

**Authors:** Tarick J. El‐Baba, Corinne A. Lutomski, Anastassia L. Kantsadi, Tika R. Malla, Tobias John, Victor Mikhailov, Jani R. Bolla, Christopher J. Schofield, Nicole Zitzmann, Ioannis Vakonakis, Carol V. Robinson

**Affiliations:** ^1^ Physical and Theoretical Chemistry Laboratory University of Oxford South Parks Rd. OX1 3QZ Oxford UK; ^2^ Department of Biochemistry University of Oxford South Parks Rd. OX1 3QU Oxford UK; ^3^ Chemistry Research Laboratory University of Oxford 12 Mansfield Rd OX1 3TA Oxford UK

**Keywords:** allosteric inhibitors, drug development, proteases, native mass spectrometry, SARS-CoV-2

## Abstract

The SARS‐CoV‐2 main protease (M^pro^) cleaves along the two viral polypeptides to release non‐structural proteins required for viral replication. M^Pro^ is an attractive target for antiviral therapies to combat the coronavirus‐2019 disease. Here, we used native mass spectrometry to characterize the functional unit of M^pro^. Analysis of the monomer/dimer equilibria reveals a dissociation constant of *K*_d_=0.14±0.03 μM, indicating M^Pro^ has a strong preference to dimerize in solution. We characterized substrate turnover rates by following temporal changes in the enzyme‐substrate complexes, and screened small molecules, that bind distant from the active site, for their ability to modulate activity. These compounds, including one proposed to disrupt the dimer, slow the rate of substrate processing by ≈35 %. This information, together with analysis of the *x*‐ray crystal structures, provides a starting point for the development of more potent molecules that allosterically regulate M^Pro^ activity.

## Graphical abstract

The SARS‐CoV‐2 main protease monomer/dimer equilibrium was characterized using native mass spectrometry. An MS‐based kinetic assay that quantifies the changes in the amounts of enzyme–substrate complex with time was used to capture M^Pro^ protease activity. Several small molecules bind non‐covalently to M^Pro^, do not compete for the active site, and slow the processing of the substrate, providing a means for optimizing potential antiviral compounds.

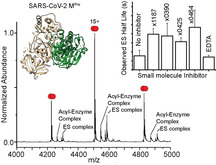

The coronavirus, SARS‐CoV‐2, is the etiological agent of the 2020 pandemic that has claimed >550 000 lives and affected more than 12 million people as of July 2020.^[1]^ Coronaviruses have long existed in nature and have made zoonotic transmission to humans. Despite the tragic and widespread effects of these sudden occurrences, we do not yet have validated anti‐viral treatments targeting coronavirus infections. SARS‐CoV‐2 packages a large RNA genome of ≈30 k bases, two‐thirds of which encodes for two polyproteins (pp1a and pp.1b). These polyproteins are processed into 16 non‐structural proteins (nsps) that are liberated from the long polypeptide chains by two viral proteases, the papain‐like protease (nsp 3) and the 3C‐like protease (nsp 5). The latter species, named the main protease M^pro^ is a cysteine protease that cleaves the viral polyproteins at eleven sites to generate twelve non‐structural proteins (nsp5‐nsp16). Included in these nsps are those involved in the replication machinery (e.g., the RNA‐dependent RNA polymerase nsp12).^[2]^ Inhibition of M^pro^ impairs the ability of the virus to replicate.

Analogous to the 2004 SARS‐CoV main protease, the functional unit of the SARS‐CoV‐2 M^Pro^ is a homodimer (Figure 1).^[3]^ Several encouraging strategies to inhibit M^Pro^ have been explored via its covalent inhibition.[^3a^, ^3b^] Non‐covalent modulators, including compounds that disrupt the dimer interface, have not been investigated to the same extent. Drugs that bind non‐covalently can often be fine‐tuned to diffuse through membranes and bind to target proteins with high affinity.^[4]^ Moreover, these species lack reactive warheads often leading to higher chemical stability than their covalent counterparts and a reduction in undesirable toxic effects due to irreversible binding to host proteins and nucleic acids. Therefore, non‐covalent compounds serve as a promising means to inhibit viral proliferation.

**Figure 1:**
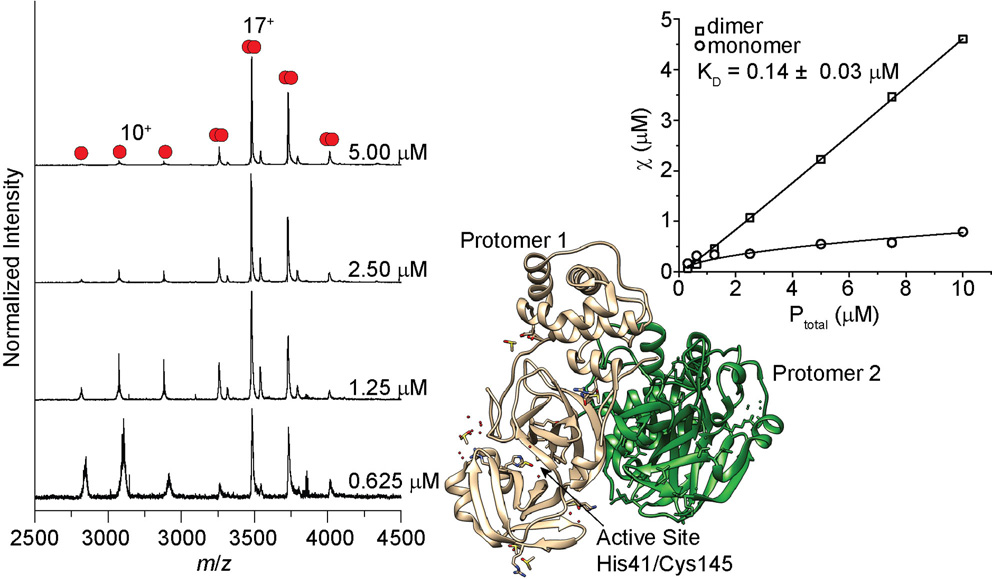
Analysis of M^Pro^ by native MS. Left: Native mass spectra for M^Pro^ at different concentrations. Right: Representative plot of mole fraction versus concentration to quantify the dissociation constant and a view from an X‐ray structure of unligated M^Pro^ (PDB ID: 6YB7).

Previous studies of the SARS‐CoV M^Pro^ dimer identified nano to micromolar dissociation constants.^[5]^ We report the dissociation constant for SARS‐CoV‐2 M^Pro^ determined using native mass spectrometry (MS), which can directly identify and quantify the relative amounts of the oligomeric state of a protein in solution.^[6]^ To probe the monomer/dimer equilibrium we recorded native mass spectra over a range of M^Pro^ concentrations from 0.313 to 10.0 μM in an aqueous buffer containing 200 mM ammonium acetate (pH 7.4). At a protein concentration of 5 μM, two well‐resolved charge state distributions are readily identified. The high‐abundant signals centered at the 17^+^ charge state correspond to a deconvoluted mass of 67 591±0.5 Da, consistent with the expected sequence mass of dimeric M^Pro^ (67 592 Da). A minor peak series between *m*/*z*≈2750 and 3500, is also observed. These signals, centered at a 10^+^ charge state, correspond to a deconvoluted mass of 33 795±3 Da, in excellent agreement with the mass of monomeric M^Pro^ with wildtype N‐ and C‐termini (33 796 Da). As the concentration of protein is decreased in a series of stepwise dilutions from 5.0 μM to 0.625 μM, the signals corresponding to the M^Pro^ monomer increase concurrent with a decrease in the peak intensities assigned to the dimer.

To extract a monomer/dimer equilibrium constant we performed measurements in triplicate at seven different protein concentrations (from 0.325 to 10 μM) and plotted the mole fraction of each species as a function of total protein concentration (Figure 1). Excellent agreement was found between the measured values and a monomer/dimer equilibrium binding model (see Experimental section in the Supporting Information) providing high confidence in the derived dissociation constant, *K*_d_=0.14±0.03 μM. This compares with an estimated *K*_d_≈2.5 μM reported recently determined using analytical ultracentrifugation.^[3b]^ The difference between the estimated value and our measurement may originate from the different buffer conditions and temperatures (20 mM Tris, 150 mM NaCl at 20 °C versus 200 mM ammonium acetate at ≈26 °C used here) both of which could perturb the monomer/dimer equilibrium. Nevertheless, we conclude that, analogous to SARS‐CoV M^Pro^, the SARS‐CoV‐2 variant has a high propensity to form dimers.

Following the deposition of high‐resolution SARS‐CoV‐2 M^Pro^ structures to the protein databank,[^3a^, ^3b^] a fragment screen was released providing structural insight towards 96 candidates that bind M^Pro^.^[3c]^ Three of these candidates were identified as binding to the dimer interface; 23 non‐covalent and 48 covalent hits were found to bind the active site. We focused our study to those small molecules that bind non‐covalently to M^Pro^ and considered their potential to both destabilize the dimer and modulate substrate cleavage. We identified four candidates, from the subset that were immediately available to us, due to their binding properties: three bind to the solvent exposed surface (x0390, x0425, and x0464) and one binds within the dimer interface (x1187). We incubated each species with 5 μM M^Pro^ at a ligand concentration range of 1 to 100 μM (0.125‐20‐fold molar excess). Following incubation for 30 mins we recorded mass spectra to investigate their effect on the monomer/dimer equilibrium. x0390, x0425, and x0464 showed no appreciable perturbation of the monomer/dimer ratios (Figure S1). However, the effect of x1187 on dimerization was clearly apparent (Figure 2). In the absence of x1187, under the solvent conditions used to dissolve the small molecules, the charge state distribution is centered at 15^+^ reinforcing the notion that M^Pro^ is predominantly a dimer under a range of solution conditions. Following the addition of a 10‐fold molar excess of x1187 the peaks assigned to monomers, centered at the 10^+^ charge state, are observed to increase. After addition of a 20‐fold molar excess of x1187 the fractional abundance of the monomer peaks increases further to ≈15 %. Saturation transfer difference nuclear magnetic resonance spectroscopy studies indicate the small molecules bind tightly to M^Pro^ in solution.^[7]^ The absence of peaks in the mass spectrum for the ligand‐bound forms of M^Pro^ likely results from the labile nature of small‐molecule binding upon transit to the gas‐phase. Moreover, comparing ligand bound species via mass spectrometry would require a different set of parameters to those optimized to monitor the monomer/dimer equilibrium. Our experiments allow us to conclude however that x1187, while bound in solution, disrupts the dimeric form of M^Pro^.

**Figure 2:**
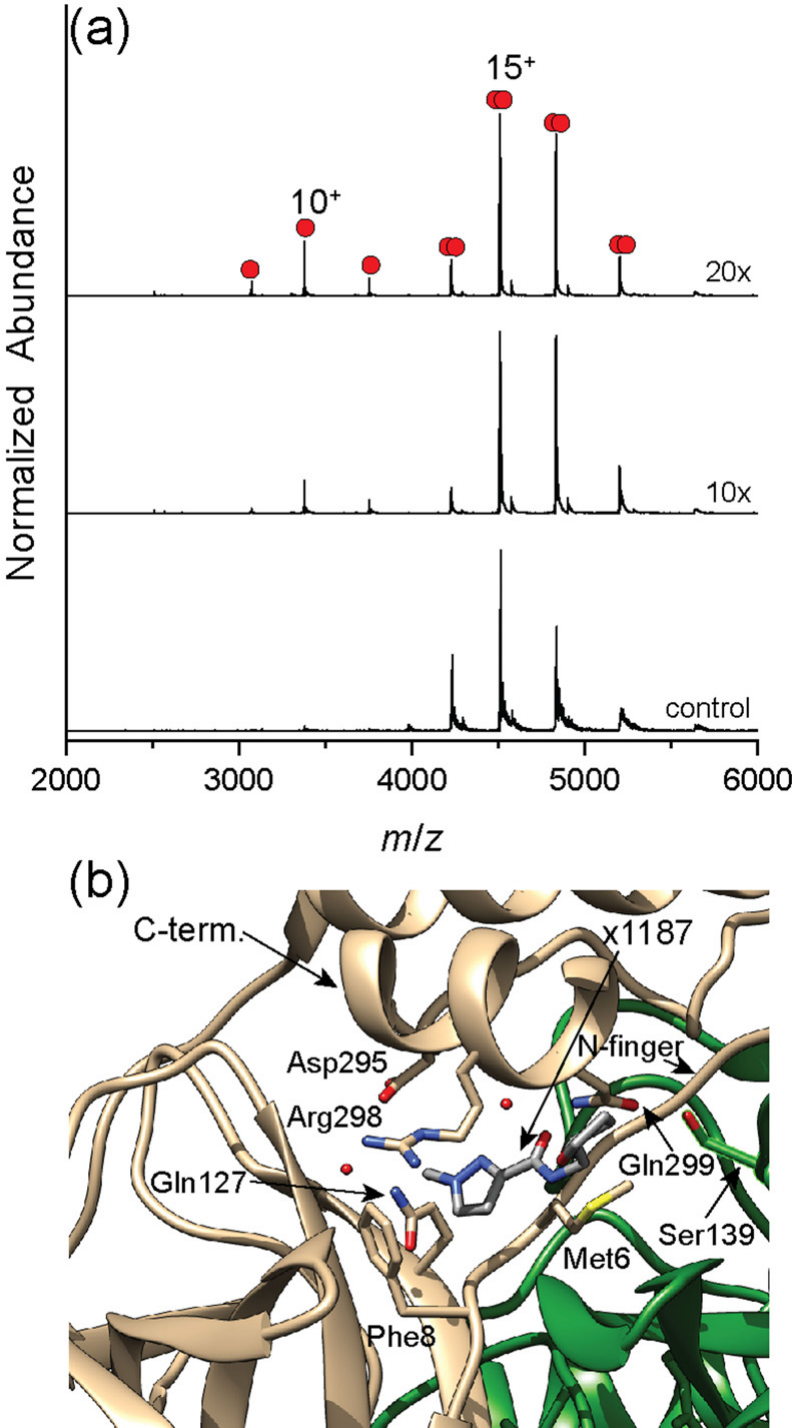
a) Native mass spectra of 5 μM M^Pro^ with the addition of different molar equivalents of x1187. To maintain similar solution conditions to the samples containing x1187, the control contains 10 % DMSO. b) Detailed view of dimer interface where x1187 binds (PDB ID: 5FRA).

Inspection of the crystal structure identifies the likely origin of this destabilization; x1187 rests across the dimer interface, packing into a hydrophobic pocket partially comprised of key residues Met6 and Phe8 on the N‐finger (Figure 2). The long axis of x1187 rests along the C‐terminal helix of protomer 1 and is proximal to Ser139 on protomer 2, a critical residue for proper function and assembly.^[8]^ The position of x1187 is important as it rests in a pocket crucial to formation of a dimer with a productive topology,^[9]^ prompting us to consider whether or not this, or the other small molecules that bind non‐covalently, mediate catalysis.

To identify the ability for these molecules to mediate the proteolytic activity of M^Pro^ we first characterized the substrate turnover in the absence of small molecules using an MS‐based kinetic assay. Rather than using a simple peptide cleavage assay, which here we found to be unreliable due to the variability in the ionization of the peptides generated, we elected to quantify changes in observable enzyme‐substrate complexes as a function of time. These complexes are notoriously difficult to isolate and quantify as they are turned over rapidly; but as they transition from solution to the gas phase, they are effectively “flash‐frozen”. Quenching the reaction enables interrogation of these transient species captured in the MS instrument.^[10]^ A mass spectrum collected 30 s after initiating the cleavage reaction is shown (Figure 3). Several satellite peaks are observed alongside the main charge state series, one of which corresponds to the enzyme substrate complex with a mass of 68 781±2 Da, in agreement with dimeric M^Pro^ bound to a single 11‐mer substrate (1192 Da). We also identified a series of peaks with a deconvoluted mass of 68 188±0.5 Da, consistent with the acyl‐enzyme complex that is formed by reaction of the nucleophilic Cys145 with the scissile Q‐S peptide bond of the substrate to give a covalent TSAVLQ‐enzyme complex of mass 68 192 Da (Figure 3). A summary of the M^Pro^‐substrate complexes we identified are listed in Table 1.

**Figure 3:**
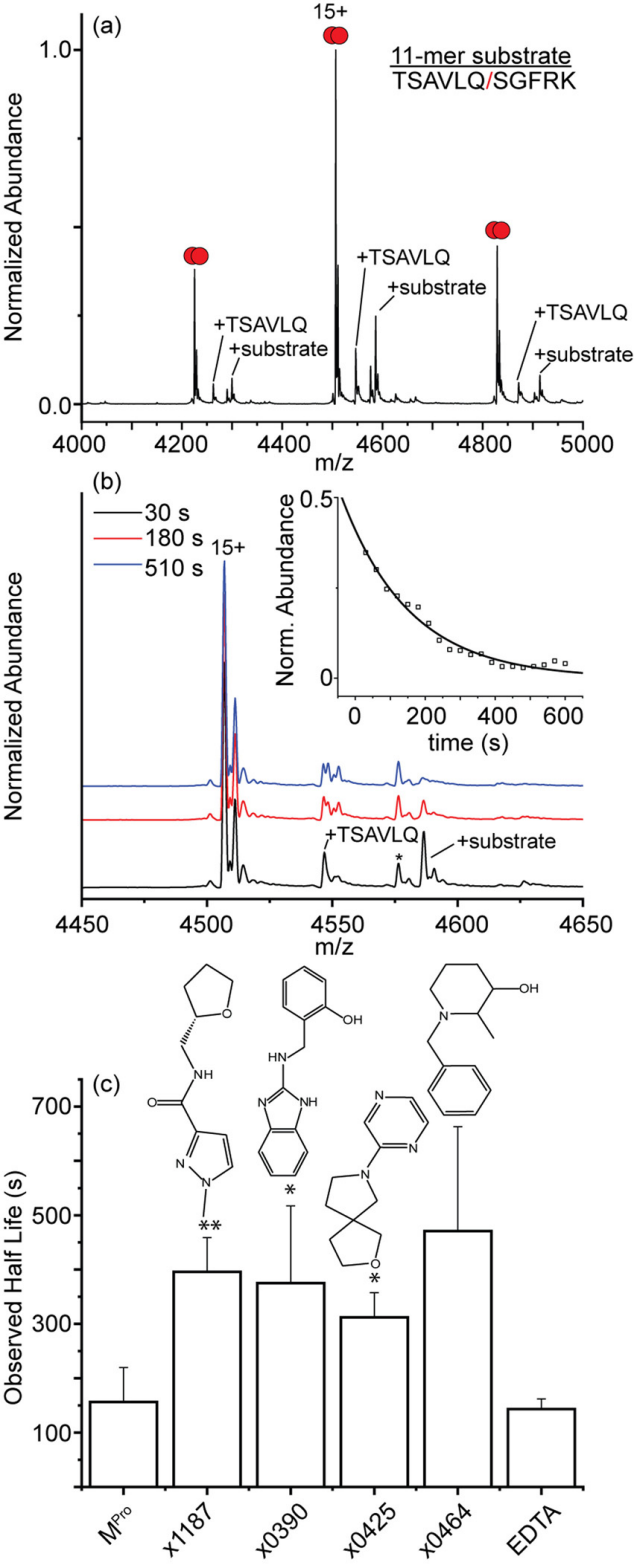
a) Native mass spectrum for 5 μM M^Pro^ with 50 μM of the 11‐mer substrate at *t*=30 s. Peaks labelled TSAVLQ and +substrate indicate acyl‐enzyme complex and the non‐covalent enzyme‐substrate complex, respectively. b) mass spectra for the 15+ charge state at three representative times along the substrate cleavage reaction. Satellite peaks adjacent to the 15+ charge state are consistent with oxidation of between 4 and 8 of the ten methionine residues (+16 Da,). Peaks marked with asterisk corresponds to an impurity (+1042 Da). Inset: plot of the relative abundance of the enzyme‐substrate complex as a function of time. Solid line indicates the fit to a unimolecular kinetics model. c) Bar plot summarizing half‐lives of the enzyme‐substrate complex in the presence of different small molecules. Error bars represent standard deviation (*n*=3 independent replicates). *p<0.05, **p<0.001 (to M^Pro^ values). Representative mass spectra and kinetic plots for each dataset are shown in Figure S4.

**Table 1:** M^Pro^ and M^Pro^‐substrate complexes identified by native MS

Species	Measured Mass [Da]^[a]^	Expected Mass [Da]	Δmass [Da]
M^Pro^ (Monomer)	33 795±3	33 796	−1
M^Pro^ (Dimer)	67 591±0.5	67 592	−1
Enzyme‐substrate complex	68 781±2	68 784	−2
Enzyme‐TSAVLQ complex	68 188±0.5	68 192	−4

[a] Uncertainty in deconvoluted mass determined using at least three charge states.

Interestingly, we did not observe any peaks corresponding to monomeric M^Pro^ bound to the 11‐mer substrate or acyl‐enzyme complex (Figure S2). We considered several reasons for the absence of signals for substrate‐bound monomers. One possibility is that the monomers have enhanced activity compared to the dimer. The timescale we measure for substrate cleavage by the dimer is on the order of minutes. Thus, if monomers were to bind and process substrates the monomer activity would need to be enhanced by ≈100‐fold (complete turnover by ≈6 s), otherwise we would readily capture monomer‐substrate complexes. This is unlikely, as other assays have indicated that monomeric M^Pro^ is inactive.^[5c]^ Our results indicate that monomers are not only inactive but also they do not bind the 11‐mer substrate with high affinity.

An expansion of the 15^+^ charge state at three representative time points reveals that as time evolves, the signal for the enzyme‐substrate complex at 4586 *m*/*z* is depleted (Figure 3). At our longest timepoint (10 min), the signals for the enzyme‐substrate complex are no longer present in detectable quantities indicating that by this time the substrate 11‐mer has been proteolyzed and the products released (Figure S3). A plot of the relative abundance of the decay of the enzyme‐substrate complex versus reaction time reveals that the decay is exponential (Figure 3). The experimental data can be readily fit to a single‐step unimolecular kinetics model, providing a relatively straightforward means to compare half‐life changes in the presence of inhibitors. A plot comparing the half‐lives obtained by incorporating the 20‐fold molar excess of the different small molecules into the reaction mixtures is shown (Figure 3 and S4). We note that the presence of these small molecules increases the lifetime of the enzyme‐substrate complex, in some cases by as much as ≈40 %.

As control experiments, we first characterized the propensity for substrate turnover using a potent inhibitor IPA3 that covalently modified Cys145 and found no appreciable changes in substrate turnover, formation of the enzyme‐substrate complex, or acyl‐enzyme complex (Figure S5). We also characterized the kinetic behavior of M^Pro^ in the presence of 50‐fold molar excess of EDTA. We found no appreciable change in the depletion of the enzyme‐substrate complex, suggesting that trace amounts of divalent metal cations, (e.g., Zn^2+^) when added in isolation, are not modulators of M^Pro^ activity.^[11]^

All of the small molecule candidates tested inhibit M^Pro^ proteolytic activity and bind to locations distant from the active site (Figure 4). Specifically, x1187 binds at the dimer interface and x0390, x0425, and x0464 bind to solvent‐exposed pockets remote form the active site. While x1187 disrupts the dimer, its proximity to key residues in the vicinity of the N‐finger may also allow it to intervene with the N‐finger in regulating activity^[9]^ and allows us to conclude that all of these fragments inhibit substrate cleavage via allosteric regulation.

**Figure 4:**
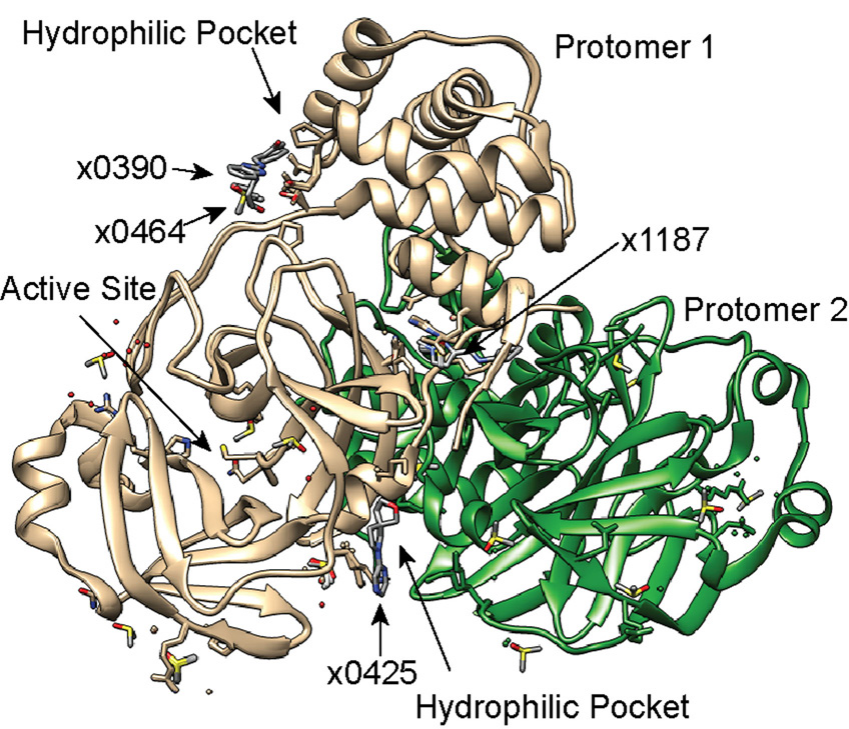
Superimposition of views of *x*‐ray structures for unligated M^Pro^ (PDB 6YB7) and M^Pro^ bound to x1187 (PDB 5RFA), x0390 (PDB 5REC), x0464 (PDB 5REE), and x0425 (5RGJ).

More generally, the therapeutic potential of these, or analogous small molecules, can be readily assessed through the dual MS approaches described here: probing the monomer/dimer equilibrium and measuring efficacy in reducing substrate turnover rates. These relatively straightforward measurements delineate the mechanism of action of these allosteric regulators—either via disruption of the dimer interface or reversible non‐competitive binding, distal from the active site. Overall, in addition to highlighting new ways of assaying these small molecules, the results provide a starting point for lead optimization chemistry with the ultimate goal of deactivating SARS‐CoV‐2 M^Pro^ and reducing the burden of the ongoing COVID‐19 pandemic.

## Conflict of interest

The authors declare no conflict of interest.

## Supporting information

As a service to our authors and readers, this journal provides supporting information supplied by the authors. Such materials are peer reviewed and may be re‐organized for online delivery, but are not copy‐edited or typeset. Technical support issues arising from supporting information (other than missing files) should be addressed to the authors.

Supplementary
